# Transactional Sex between Men and Its Implications on HIV and Sexually Transmitted Infections in Nigeria

**DOI:** 10.1155/2017/1810346

**Published:** 2017-08-06

**Authors:** Eniola A. Bamgboye, Titilope Badru, Afolabi Bamgboye

**Affiliations:** ^1^Department of Epidemiology and Medical Statistics, College of Medicine, Faculty of Public Health, University of Ibadan, Ibadan, Oyo State, Nigeria; ^2^Society for Family Health, 8 Port Harcourt Crescent, Area 11, Garki, Abuja, Nigeria; ^3^Folbam Health Research and Data Management Limited, 64 Oyo Road, Samonda, Ibadan, Oyo State, Nigeria

## Abstract

**Introduction:**

Men who have transactional sex with men (MTSM) are known to be at higher risk for HIV and sexually transmitted infections (STIs). This study explored the risk factors associated with STI symptoms and HIV prevalence among men who have transactional sex with men in Nigeria.

**Methods:**

In 2014, a cross-sectional study, using respondent driven sampling technique, was carried out to recruit 3,172 MSM across eight states in Nigeria. Relevant information on sociodemographic characteristics, sexual behaviors, and self-reported symptoms of STI was obtained. Bivariate and multivariate analysis was performed to identify risk factors for STI symptoms and HIV.

**Results:**

38.2% of the MSM were involved in transactional sex. Prevalence of self-reported STI symptoms was higher among MTSM than other MSM, while HIV prevalence was higher among other MSM than MTSM. Identified factors associated with STI symptoms and HIV among MSTM were being single, alcohol consumption, oral sex, and history of rape by a male partner.

**Conclusion:**

Sexually transmitted infections are a significant challenge to men who have transactional sex with men. Adolescents and single men are more at risk of these infections. Youth empowerment needs to be invested on to avoid increased risk among these groups of people.

## 1. Background

Transactional sex (TS) is generally defined as the trading (buying or selling) of sex for material benefit (i.e., exchanging money, drugs, food, shelter, or other items for sex). Various studies have reported increased prevalence of TS among men who have sex with other men (MSM). Engagement in TS occurs along a spectrum of participation ranging from casual, infrequent encounters, to continual professional exchange.

Transactional sex between men frequently involves anal intercourse which, if unprotected, carries a high risk of transmission of sexually transmitted infections for the receptive partner, and a significant risk for the insertive partner. Male-male sex is often initiated during adolescent years and is very common in the repertoire of adolescent sexual experimentation, thus making them more vulnerable to risky sexual behaviors and perpetrators are seen as key vectors for HIV transmission [1, 2]. Research has also shown that men who sell sex are more likely than other men who have sex with men (MSM) to engage in unprotected anal sex with their non-TS male and female partners, thus putting them at a higher risk of these infections [3, 4].

Nigeria ranks second in the world in terms of the number of people living with HIV/AIDS; the adult HIV prevalence increased from 1.8% in 1991 to 5.8% in 2001, before dropping to 4.4% in 2005 and further to 3.3% by 2014 [5]. From the 2010 national HIV seroprevalence study, 4.1% was obtained for the general population, while MSM had prevalence above 13% [6]. This trend was also seen in a study conducted in three cities in Nigeria where MSM had a prevalence four to ten times higher than the general population [7]. Twenty-three percent of new cases of HIV infection in the same year were attributed to high risks groups including MSM, female who sell sex (FWSS), and people who inject drugs (PWID). A recent report from the 2014 Integrated Behavioural Biological Survey (IBBS) conducted among key populations in Nigeria revealed that MSM had the highest prevalence of HIV (20.1%) followed closely by brothel-based female who sell sex (19.8%) [8].

A considerable number of studies have reported prevalence of HIV among MSM in Nigeria, but few studies exist in this setting that have documented practices and motivating forces underlying men's transactional sexual relationships with other men and its implications on sexually transmitted infections including HIV [5]. Therefore this study aimed to determine the prevalence and explore the risk factors associated with STI symptoms and HIV among men who have transactional sex with men in Nigeria.

## 2. Methodology

### 2.1. Study Setting

Nigeria, the largest country in the western region of Africa, is a federation of 36 states and the Federal Capital Territory (FCT) with 774 administrative units referred to as Local Government Areas. The country has an estimated population of 182,200,000 by the World Bank in 2015 [6]. More than half of Nigerians (54.4%) live in poverty in spite of the huge revenues accruing from oil and gas. The country is composed of more than 250 ethnic groups, with Yoruba, Igbo, and Hausa being the most dominant.

### 2.2. Study Design

A cross-sectional study, using a respondent driven sampling technique was carried out in September, 2014, to recruit men who have sex with men (MSM) in eight major cities with relatively higher HIV prevalence in Nigeria. The cities were Kano, Lagos, Cross River, Enugu, Kaduna, Rivers, Oyo, and the Federal Capital Territory [8]. A total of five seeds were initially selected through nongovernmental organization networks that historically provide support and services for MSM community. Seeds were selected to reflect the diversity within the MSM population (i.e., age, sexual identity, ethnicity, geographic area, and socioeconomic status). Each seed was given three coupons and incentives ($5) to further recruit other MSM.

### 2.3. Data Collection

An interviewer administered semistructured questionnaire was used to obtain information from a total of 3,172 MSM who were 15 years old and above. Information such as sociodemographic characteristics, sexual behaviors, and symptoms of STI was obtained. HIV test was carried out using the Rapid Test Kit and status was confirmed with Western Blot. Transactional sex was defined as ever having any sexual intercourse involving paying (money and gifts) for sex or receiving any money or gifts in exchange for sex. Respondents were asked whether they had any of the following STI symptoms in the last 6 months: genital/anal discharge, genital/anal ulcers, genital/anal sores, and genital/anal warts. Written informed consent was obtained from the respondents and they were given a copy to keep.

### 2.4. Data Analysis

Estimates of MSM were obtained using the RDSAT software and SPSS v.20 was used to explore the prevalence and factors associated with STI symptoms and HIV among MTSM after weighting the data. The RDSTAT software weights data for unequal probabilities of selection and networking. Frequencies and proportions were obtained for categorical variables, while bivariate analysis was done using the chi-square test. Multivariate analysis using logistic regression was carried out to identify independent predictors of STI symptoms and HIV among MTSM. Only variables at 10% level of significance were included in the logistic regression model. All statistical significance was set at 5%.

## 3. Results 

A total of 3,172 men who have sex with men were included in this study.  Table 1 shows the sociodemographic characteristics of men who have sex with men by their involvement in transactional sex. The prevalence of transactional sex among MSM was found to be 38.2% with the highest proportion being seen among the age group of 15–19 years (44.7%) and those with at least secondary education (73.9%), *p* < 0.05. The proportion of MSM who were involved in transactional sex was also higher among men who were married as compared to men who were not married, while only 29.5% married men who were not living with heterosexual partners were involved in transactional sex. Among the states in which this study has been conducted, Kano (61.9%) had the highest proportion of MTSM followed by Kaduna (52.2%) and Oyo (51.5%) states, with the least being in the Federal Capital Territory (22.7%).

In addition, a higher proportion of MSM used condom consistently (41.9%); those involved in oral sex with other men (47.4%) and those who used lubricant at last anal sex (40.1%) were involved in transactional sex. However, a higher proportion of MSM who consumed alcohol daily (56.0%), injected drugs (70.0%), and practiced both insertive and receptive anal sex (46.9%) were involved in transactional sex with other men (Table 1).

### 3.1. Prevalence of STI Symptoms and HIV among Men Who Engage in Transactional Sex with Other Men

**Figure 1:**
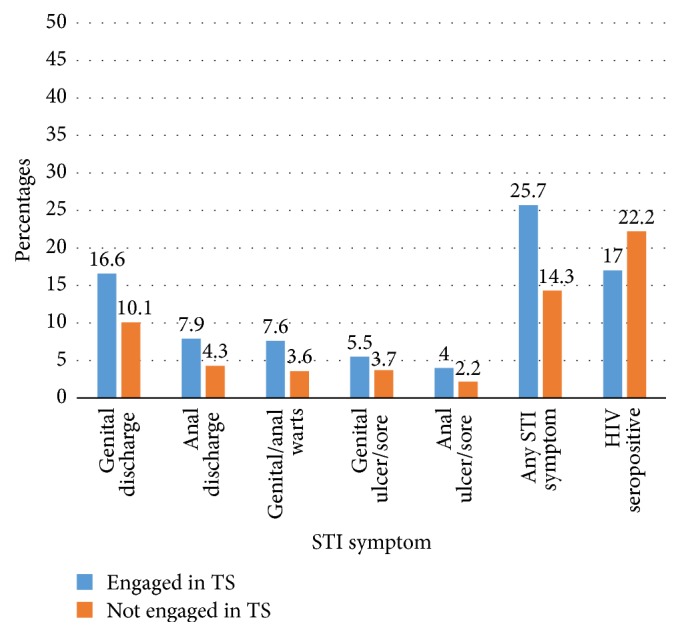
Proportion of MSM who have sexually transmitted symptoms and HIV by engagement in transactional sex (TS).

Self-reported STI symptoms were found to be higher among MTSM (25.7%) compared with 14.3% among men who do not engage in transactional sex. The most common STI symptom was genital discharge (16.6%) followed by anal discharge (7.9%), genital/anal warts (7.6%), genital ulcer/sore (5.5%), and anal ulcer/sore (4.0%). Conversely, HIV prevalence was higher among men who do not engage in transactional sex with other men (22.2%) compared to 17.0% among MTSM (Figure 1()).

Table 2 revealed that respondents with primary education had the highest proportion of MTSM with STI symptoms (37.1%) compared to those with secondary (24.6%) or tertiary (27.5%) education. However, this was not statistically significant (*p* > 0.05). MTSM who were not married but living with heterosexual partner also had the highest proportion with STI symptoms (40.0%) compared to married MSM living with their spouse (18.9%) or living with other sexual partner (20.0%) (*p* < 0.05). So also, MTSM who were involved in oral sex with other men (29.9%), practiced both insertive and receptive sex (28.8%), consumed alcohol daily (39.6%), and ever experienced rape by a male partner (32.5%) had a significantly higher proportion with STI symptoms (*p* < 0.05).

Information regarding HIV status and associated factors among MTSM is also presented in the fourth column of Table 2. HIV prevalence was highest among MTSM aged 25 years and above (25.9%) as compared to 10% in those aged 15–19 years (*p* < 0.05). MTSM with tertiary education (20.2%), who were not married but living with heterosexual partner (22.6%), were engaged in oral sex (18.3%), practiced receptive anal sex only (19.0%), injected drugs (20.0%), and ever experienced rape by a male partner (18.5%) had higher HIV prevalence. However, these variables were not found to be statistically significant (*p* > 0.05). On the other hand, MTSM who consumed alcohol less than once a week (23.0%) or never consumed alcohol (17.1%) had higher proportions with HIV compared to those who consumed alcohol daily (8.9%) (*p* < 0.05).

### 3.2. Factors Associated with STI Symptoms and HIV among Men Who Engage in Transactional Sex with Other Men

The predictors of STI symptoms and HIV status are presented in Table 3. MTSM who were aged 25 years and above were 4 times more likely to have HIV infection (AOR: 4.1, 95% CI: 2.9–5.8), while those that were not married but living with heterosexual partner were about 2 times more likely to have HIV infection (AOR: 1.87, 95% CI: 1.0–3.4). Also MTSM who do not have oral sex with men were about 2 times less likely to have STI symptoms (AOR: 0.51, 95% CI: 0.4–0.6) and HIV (AOR: 0.77, 95% CI: 0.6–0.9). Alcohol consumption was also found to be an independent predictor as those who had never drank were 2 times less likely to have STI symptoms (AOR: 0.55, 95% CI: 0.4–0.7) but about 4 times more likely to have HIV (AOR: 3.90, 95% CI: 2.2–6.6). Lubricant (water based type) use was also found to be a predictor of HIV infection as those who did not use lubricant at last anal intercourse were 1.5 times less likely to have HIV infection (AOR: 0.65, 95% CI: 0.5–0.8), while those who had not been forced to have anal intercourse with a male partner were 1.7 times less likely to have STI symptoms (AOR: 0.58, 95% CI: 0.4–0.7). Furthermore, the type of anal sex was found to be an independent predictor of STI symptoms and HIV, respectively, as those that engage in both insertive and receptive anal sex were about 1.5 times more likely to have STI symptoms (AOR: 1.3, 95% CI: 1.1–1.6) and HIV infection (AOR: 1.4, 95% CI: 1.1–1.8).

## 4. Discussion

The prevalence of sexually transmitted infections including HIV has been reported to be higher among sex workers and their clients than the general population. It is not surprising then that sex work plays an important role in sexually transmitted infections. This study highlights the high prevalence of transactional sex among men who have sex with men and the significant association with STI symptoms in some Nigerian cities. The finding that about a fourth of MSM interviewed in this study engaged in transactional sex with other men are in consonance with several studies reported from Latin America, Kenya, and Nigeria [9–12], though it is slightly lower than the 50% reported in a systematic review of transactional sex including sub-Saharan Africa [10]. This study also reveals that transactional sex was more among younger men aged 15–19 years and this is similar to a good number of studies including a study done in South America that reported age at transactional anal sexual debut of 15.5 years [9, 13–15].

On the other hand, studies have reported HIV to be associated with transactional sex among men, but findings from this study showed a slightly higher HIV prevalence among men who have sex with men but do not engage in transactional sex [10, 12, 16–18]. This could be due to the self-perceived risk of contracting HIV and thus initiation of protective measures.

In view of the high biological risk associated with anal intercourse, unprotected anal intercourse is a risky behavior that has been implicated in HIV transmission while the use of condoms has been shown to provide protection against sexually transmitted infections. However, this study reported that just about a quarter of MSM use condoms consistently and this was even lower among men who engage in transactional sex. The findings were in contrast to some previous studies carried out among MSM in Nigeria irrespective of engagement in transactional sex which reported 62.5% in Lagos State in 2011 and 43.4% in three cities of Nigeria in 2010. This dissimilarity might have been observed because of the larger number of respondents interviewed and the categorization of MSM into those involved in transactional sex or not. Worthy of note is the fact that this study found a relatively high proportion of MSM who use lubricant but this was negatively associated with HIV status. MTSM who do not use lubricants were less likely to be infected with HIV probably because use of lubricants could increase the practice of riskier sexual behavior since they are involved in economic sex transactions. Such men who used lubricants could also have thought that the lubricants would protect them from infection and so did not use condoms whenever they used lubricants.

Sexually transmitted infection symptoms were seen to be present in a significantly higher proportion of MTSM than non-MTSM. This shows that MTSM are more likely to develop STIs due to their engagement in riskier sexual behavior which might be due to the fact they are being paid or pay for sex; thus their negotiating power for condom use is reduced. Furthermore, behaviors that predicted MTSM having STI symptoms as obtained from this study include alcohol consumption, oral sex with other men, bisexuality, and history of rape which are in line with other studies reported in the same setting [19–22].

In conclusion, prevalence of STI symptoms among men who engage in transactional sex with other men was high. The study illustrates the importance of social vulnerability in driving the HIV epidemic. Programs to empower young MSM reduce social vulnerability and other structural barriers including negotiating for safe sex are critical to reduce transmission of sexually transmitted diseases.

## Figures and Tables

**Table 1:** Sociodemographic correlates of men who have sex with men by transactional status.

	Transactional sex		Total	Chi-square	*p* value
	Yes	No			
Age (years)					
15–19	294 (44.7)	363 (55.3)	657 (20.7)		
20–24	577 (39.6)	880 (60.4)	1457 (45.9)	29.1	<0.001
25 and above	341 (32.2)	717 (67.8)	1058 (33.4)		
Education					
None/primary	35 (34.3)	67 (65.7)	102 (3.2)		
Secondary	886 (40.2)	1320 (59.8)	2206 (69.5)	11.7	<0.001
Tertiary	291 (33.7)	573 (66.3)	864 (27.2)		
Religion					
None	8 (44.4)	10 (55.6)	18 (0.6)		
Christianity	798 (35.8)	1433 (64.2)	2231 (70.3)		
Islam	406 (44.0)	517 (56.0)	923 (29.1)	18.9	<0.001
Marital status					
Married living with spouse	74 (49.7)	75 (50.3)	149 (4.7)		
Married living other sexual partner	5 (45.5)	6 (54.5)	11 (0.3)		
Married not living with sexual partner	5 (29.4)	12 (70.6)	17 (0.5)	26.0	<0.001
Not married living with sexual partner	105 (51.0)	101 (49.0)	206 (6.5)		
Not married, not living with sexual partner	1023 (36.7)	1766 (63.3)	2789 (87.9)		
State					
Cross River	143 (31.4)	312 (68.6)	455 (14.3)		
Enugu	210 (46.8)	239 (53.2)	449 (14.2)		
FCT	136 (22.7)	463 (77.3)	599 (18.9)		
Kaduna	247 (52.2)	226 (47.8)	473 (14.9)	212.2	<0.001
Kano	60 (61.9)	37 (38.1)	97 (3.1)		
Lagos	84 (40.4)	124 (59.6)	208 (6.6)		
Oyo	212 (51.5)	200 (48.5)	412 (13.0)		
Rivers	120 (25.1)	359 (74.9)	479 (15.1)		
Total	1212 (38.2)	1960 (61.8)	3172 (100.0)		

**Table 2:** Factors associated with STI symptoms and HIV infection among men who engage in transactional sex with other men.

Selected characteristics	STI symptoms	Total	HIV positive	Total
Age				
15–19 yrs	75 (25.5)	294 (24.3)	25 (10.0)	250 (100.0)
20–24 yrs	147 (25.5)	577 (47.6)	72 (15.4)	467 (46.6)
25 yrs and above	89 (26.1)	341 (28.1)	74 (25.9)	286 (28.5)
	*χ*^2^ = 0.04	*p* = 0.97	*χ* ^2^ = 25.4	*p* < 0.001
Education				
None/primary	13 (37.1)	35 (2.9)	6 (17.1)	35 (3.5)
Secondary	218 (24.6)	886 (73.1)	118 (16.1)	735 (73.3)
Tertiary	80 (27.5)	291 (24.0)	47 (20.2)	233 (23.2)
	*χ* ^2^ = 3.44	*p* = 0.17	*χ* ^2^ = 2.12	*p* = 0.34
Marital status				
Married living with spouse	14 (18.9)	74 (6.1)	10 (14.5)	69 (6.9)
Married living other sexual partner	1 (20.0)	5 (0.4)	0 (0.0)	4 (0.4)
Married not living with sexual partner	2 (40.0)	5 (0.5)	1 (20.0)	5 (0.5)
Not married living with sexual partner	42 (40.0)	105 (8.7)	21 (22.6)	93 (9.3)
Not married, not living with sexual partner	252 (24.6)	1023 (84.4)	139 (16.7)	832 (83.3)
	*χ* ^2^ = 14.27	*p* < 0.001	*χ* ^2^ = 3.25	*p* = 0.51
Consistent condom use				
Yes	85 (25.2)	337 (27.8)	121 (17.0)	708 (70.6)
No	226 (25.8)	875 (72.2)	50 (16.9)	295 (29.4)
	*χ* ^2^ = 0.04	*p* = 0.82	*χ* ^2^ = 0.00	*p* = 0.95
Oral sex with another man				
Yes	232 (29.9)	776 (64.0)	119 (18.3)	650 (64.8)
No	79 (18.1)	436 (36.0)	52 (14.7)	353 (35.2)
	*χ* ^2^ = 20.2	*p* < 0.001	*χ* ^2^ = 2.06	*p* = 0.15
Alcohol consumption				
Daily	57 (39.6)	144 (11.9)	10 (8.6)	116 (11.6)
At least once a week	69 (27.4)	252 (20.8)	32 (14.7)	217 (21.6)
Less than once a week	77 (23.4)	329 (27.1)	57 (23.0)	248 (24.7)
Never	108 (22.2)	487 (40.2)	72 (17.1)	422 (42.1)
	*χ* ^2^ = 19.0	*p* < 0.001	*χ* ^2^ = 12.8	*p* < 0.001
Injected drugs				
Yes	2 (28.6)	7 (0.6)	1 (20.0)	5 (0.5)
No	309 (25.7)	1205 (99.4)	170 (17.1)	997 (99.5)
	*χ* ^2^ = 0.37	*p* = 0.82	*χ* ^2^ = 0.23	*p* = 0.88
Lubricant use				
Yes	257 (25.7)	999 (82.4)	150 (18.5)	813 (81.1)
No	54 (25.4)	213 (17.6)	21 (11.1)	190 (18.9)
	*χ* ^2^ = 0.01	*p* = 0.91	*χ* ^2^ = 5.95	*p* < 0.001
Ever been forced to have sex with male partner				
Yes	120 (32.5)	369 (30.4)	59 (19.0)	311 (31.0)
No	191 (22.7)	843 (69.6)	112 (16.2)	692 (69.0)
	*χ* ^2^ = 13.0	*p* < 0.001	*χ* ^2^ = 1.17	*p* = 0.27
Type of anal sex				
Insertive only	67 (24.2)	277 (22.9)	37 (15.5)	238 (23.7)
Receptive only	49 (19.0)	258 (21.3)	38 (19.0)	200 (19.9)
Both	195 (28.8)	677 (55.9)	96 (17.0)	565 (56.3)
	*χ* ^2^ = 9.83	*p* < 0.01	*χ* ^2^ = 0.92	*p* = 0.63
* Total*	*311 (25.7)*	*1212 (100.0)*	*171 (17.0)*	*1003 (100.0)*

**Table 3:** Regression coefficients and odds ratio for the predictors of STI symptoms and HIV among men who engage in transactional sex with other men.

	STI symptoms			HIV status		
	AOR	95% CI	*p* value	AOR	95% CI	*p* value
*Predictor variables*						
Age (years)						
15–19						
20–24	0.90	0.7–1.2	0.436	2.00	1.4–2.7	<0.001
25 and above	0.85	0.6–1.1	0.284	4.14	2.9–5.8	<0.001
Education						
None/primary						
Secondary	0.83	0.4–1.4	0.496	1.11	0.6–1.9	0.687
Tertiary	0.80	0.5–1.4	0.455	1.37	0.7–2.2	0.276
Marital status						
Married living with spouse						
Married living other sexual partner	1.71	0.4–7.0	0.453	2.63	0.6–11.8	0.206
Married not living with sexual partner	0.51	0.1–2.47	0.411	2.12	0.6–6.9	0.215
Not married living with sexual partner	1.40	0.8–2.36	0.199	1.84	1.0–3.3	0.047
Not married, not living with sexual partner	0.80	0.5–1.2	0.802	1.87	1.1–3.0	<0.01
Oral sex with another man						
Yes						
No	0.51	0.4–0.6	0.00	0.77	0.6–0.9	<0.02
Alcohol consumption						
Daily						
At least once a week	0.67	0.4–0.9	0.02	2.12	1.2–3.7	<0.001
Less than once a week	0.60	0.4–0.8	0.00	4.06	2.3–6.9	<0.001
Never	0.55	0.4–0.7	0.00	3.90	2.2–6.6	<0.001
Lubricant use						
Yes						
No	0.98	0.7–1.2	0.91	0.65	0.5–0.8	<0.001
Ever been forced to have sex with male partner						
Yes						
No	0.58	0.4–0.7	0.00	0.76	0.7–1.2	0.761
Type of anal sex						
Insertive only						
Receptive only	0.88	0.6–1.1	0.37	1.46	1.1–1.9	<0.001
Both	1.3	1.1–1.6	0.00	1.40	1.1–1.8	<0.001

## Additional Points

*Limitation of Study*. This study acknowledges the fact that STI symptoms were self-reported and they may not actually have been confirmed clinically by the respondents. In addition, due to the nature of the study design (cross-sectional), we cannot conclude that transactional sex leads to sexually transmitted diseases or HIV infection (or vice versa), but we can say sexually transmitted diseases including HIV infection are heightened when one engages in transactional sex.

*Key Messages*. (i) Transactional sex among men who have sex with men in Nigeria is prevalent. (ii) Sexually transmitted infection symptoms should be addressed urgently among men who have transactional sex with men. (iii) HIV infection among MSM is higher than the national average; measures to protect MSM from HIV infection should be intensified in Nigeria. (iv) Adolescents and single men are more at risk of these infections.
